# A particular bigeminy during atrial tachycardia

**DOI:** 10.1007/s12471-014-0572-6

**Published:** 2014-07-10

**Authors:** C. Buttà, A. Tuttolomondo, L. Giarrusso, A. Pinto

**Affiliations:** 1 U.O.C. Medicina Interna e Cardioangiologia, Dipartimento Biomedico di Medicina Interna e Specialistica, Università degli Studi di Palermo, Palermo, Italy; 2 U.O.C. Medicina Interna e Cardioangiologia Dipartimento Biomedico di Medicina Interna e Specialistica, Università degli Studi di Palermo, Piazza delle Cliniche n° 2, 90127 Palermo, Italy

**Fig. 1:**
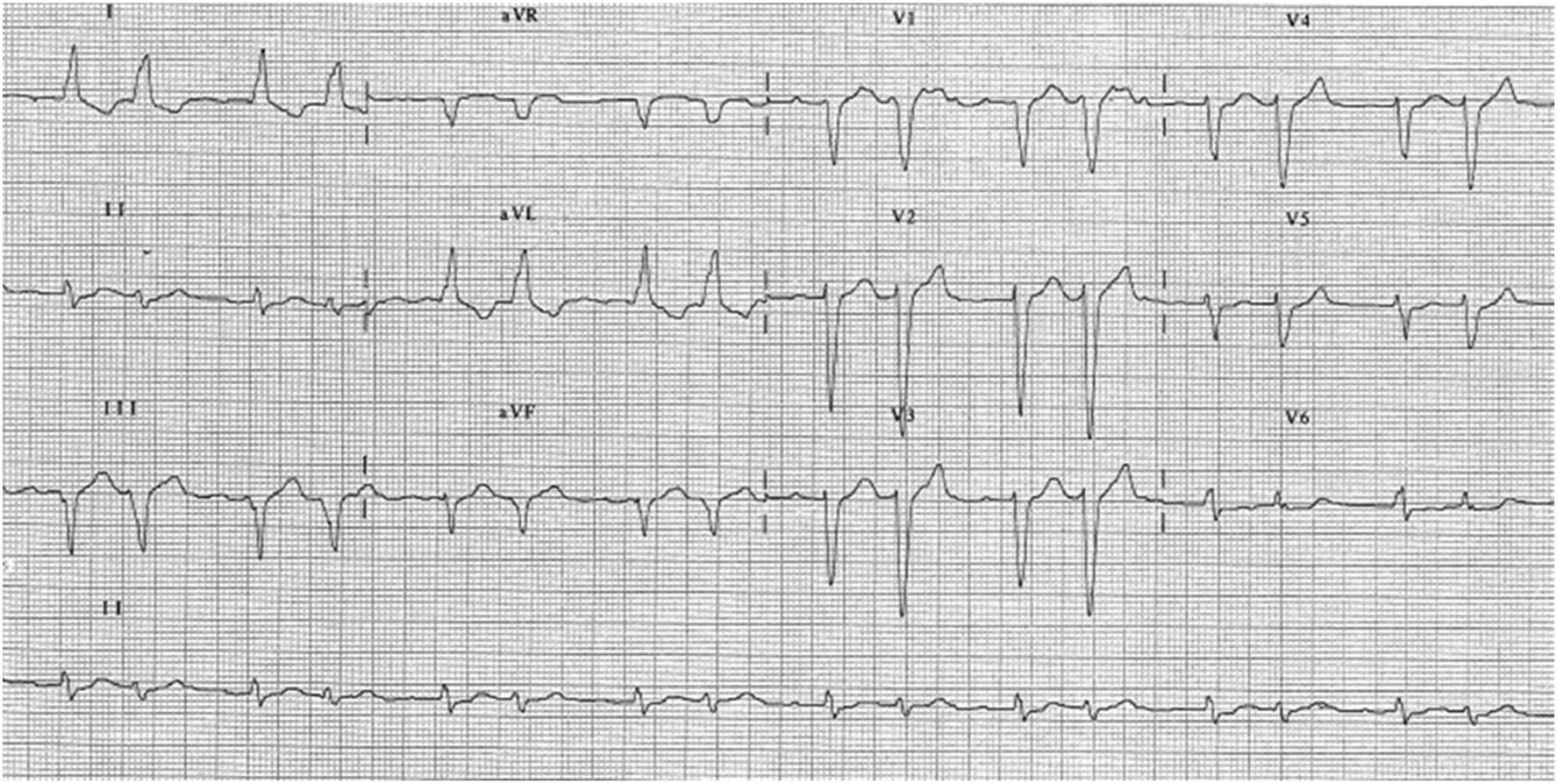
A particular bigeminy with aberrant conduction during atrial tachycardia

A 77-year-old female patient presented to the hospital’s emergency department with palpitations. The electrocardiogram shows a supraventricular bigeminy where the premature beats are due to aberrant conduction (wider and deeper S waves from lead V1 to V5 and ‘RSR’ morphology in lead V6) but the rhythm is not sinus (Fig. 1). What is the mechanism?

Answer

You will find the answer elsewhere in this issue.

